# Human prefrontal layer II interneurons in areas 46, 10 and 24

**Published:** 2015-03-30

**Authors:** Gabriel Arteaga, Efrain Buritica, Martha Isabel Escobar, Hernan Pimienta

**Affiliations:** 1 Centro de Estudios Cerebrales, Facultad de Salud, Universidad del Valle, Cali, Colombia

**Keywords:** Cerebral cortex, GABAergic Neurons, Working Memory, working memory, calcium-binding proteins, Neocortical External Granular Layer

## Abstract

**Background:**

Prefrontal cortex (PFC) represents the highest level of integration and control of psychic and behavioral states. Several dysfunctions such as autism, hyperactivity disorders, depression, and schizophrenia have been related with alterations in the prefrontal cortex (PFC). Among the cortical layers of the PFC, layer II shows a particular vertical pattern of organization, the highest cell density and the biggest non-pyramidal/pyramidal neuronal ratio. We currently characterized the layer II cytoarchitecture in human areas 10, 24, and 46.

**Objective:**

We focused particularly on the inhibitory neurons taking into account that these cells are involved in sustained firing (SF) after stimuli disappearance.

**Methods:**

Postmortem samples from five subjects who died by causes different to central nervous system diseases were studied. Immunohistochemistry for the neuronal markers, NeuN, parvalbumin (PV), calbindin (CB), and calretinin (CR) were used. NeuN targeted the total neuronal population while the rest of the markers specifically the interneurons.

**Results:**

Cell density and soma size were statically different between areas 10, 46, 24 when using NeuN. Layer II of area 46 showed the highest cell density. Regarding interneurons, PV+-cells of area 46 showed the highest density and size, in accordance to the proposal of a dual origin of the cerebral cortex. Interhemispheric asymmetries were not identified between homologue areas.

**Conclusion:**

First, our findings suggest that layer II of area 46 exhibits the most powerful inhibitory system compared to the other prefrontal areas analyzed. This feature is not only characteristic of the PFC but also supports a particular role of layer II of area 46 in SF. Additionally, known functional asymmetries between hemispheres might not be supported by morphological asymmetries.

## Abstract (es)

**Antecedentes:**

La corteza prefrontal (CPF) representa el nivel más alto de integración y control de funciones psíquicas y comportamentales. Varias patologías como autismo, desórdenes de hiperactividad, depresión y esquizofrenia se han relacionado con alteraciones de la CPF. La lámina II de las áreas que constituyen la CPF posee un patrón de organización vertical, una alta densidad celular y la mayor proporción de neuronas no-piramidal/piramidal. Sin embargo, la distribución del componente inhibitorio en estas regiones no se ha descrito. En el presente estudio nos propusimos caracterizar la lámina II de las áreas 10, 24 y 46 del humano, particularmente su componente inhibitorio teniendo en mente su participación en procesos de actividad sostenida relevantes cuando desaparece el estímulo.

**Métodos:**

Se utilizaron muestras de cinco sujetos que fallecieron por causas diferentes a enfermedades del sistema nervioso. Se tomaron secciones de las áreas 10,24 y 46 de Brodmann y se procesaron con los anticuerpos contra NeuN para determinar la población neuronal total y contra parvalminina (PV), calbindina (CB) y calretinina )CR) para analizar la población de interneuronas.

**Resultados:**

Los resultados no mostraron diferencias interhemisféricas entre las áreas. Sin embargo, las tres áreas seleccionadas son significativamente diferentes entre sí en todos los parámetros analizados. El área 46 posee la mayor densidad y tamaño de interneuronas positivas para PV.

**Conclusiones:**

La ausencia de asimetrías morfológicas no permite explicar las asimetrías funcionales. La lámina II del área 46 posee el sistema inhibitorio más poderoso. Teniendo en cuenta la arquitectura modular de las capas supragranulares, este sistema inhibitorio subyace a la actividad sostenida, eje fundamental de la memoria operativa.

## Introduction

The prefrontal cortex (PFC) represents 30% of the whole cortical surface in humans. In accordance to the proposal of a "dual origin of the cerebral cortex", in mammals, cortical areas can be traced as originating from a medio-ventro-lateral train in the olfactory cortex (paleocortex) and a medio-dorso-lateral one in the hippocampus (archicortex). These two trends converge in the lateral surface of the DL PFC at the level of Brodmann areas 8 and 46 1.

Specific alterations in the PFC result in severe behavior and cognitive disturbances such as those observed in autism 2 ^,^ 3, bipolar disorder 4; and ADHD 5. Additionally, some authors described alterations at cell and synaptic levels specifically in supragranular layers in schizophrenia 6 ^,^ 7.

The PFC is characterized based on how the two main neuronal types (pyramidal cells and interneurons) are organized and distributed in layers from the pial surface to white matter. Thus, three main subdivisions, a dorsolateral (DLPFC), orbitofrontal (OFPFC), and ventromedial (VMPFC) have been determined. In primate DLPFC, most of interneurons (65%) are located in layer II and superficial layer III. Interneurons are classically studied by the specific expression of calcium binding proteins (CBP). Accordingly, calretinin immunoreactive (CR-IR) small cells are located below layer I. Calbindin immunoreactive (CB-IR) neurons are seen in deeper regions of these layers. Additionally, a bigger and denser population of cells, parvalbumin immunoreactive (PV-IR), are identified in the superficial region of layer III. Since the acknowledgment of both specific layer location of interneurons and how these cells actually interact with pyramidal cells is required to figure out regulatory functionality in PFC, different studies have made attempts to address the issue. Nevertheless, achievements in this regard are still poor 8 ^,^ 9.

Furthermore, several cortical areas of mammals, including PFC, display a column-shaped modular organization in layers I and II. Small iterative modules interconnected by recurrent projections with excitatory intermodular activity (reverberating), which are in turn precisely regulated by a wide cohort of gabaergic interneurons, constitute the currently known description. This pattern is distinguishable from the columnar system described in other cortical layers 10 ^-^ 13.

As matter of great interest to the current work, the previous vertical-modular organization may constitute the structural basis of the phenomenon known as Sustained firing (SF) that is known to happen in the DLPFC. Sustained firing is thought to result in neural networks after the "turnoff" of an initial electric trigger. Such activity in the DLPFC of human and superior primates has been proposed as a physiological substrate for complex cognitive functions, such as working memory, sustained attention, decision taking, and in general terms the "cognitive control". All of these functions have as common the keeping up of an active representation (online) of the reality whereas goals at behavioral and cognitive levels are accomplished 14 ^-^ 16. 

On the other hand, morphological features of neurons in layer II of DLPFC are considered especially interesting among PFC subdivisions. For instance, previous morphometric studies on layer II of DLFPC have demonstrated particularly abundant neurons which are also densely packed in a small thickness. Additionally, since cell soma size of neurons is correlated with the length and geometry of dendritic and axonal processes, sizes between 80-100 µm^2 ^found in cells of layer II of DLPFC predict processes long enough to just cover two contiguous modules 17. Altogether, these features are functionally consistent to a massive but delicate integration of a large amount of afferents coming from all cortical areas and subcortical structures 18.

The present study characterized the cell organization of the layer II of the human Brodmann areas 10, 24, and 46; which are representative of OFPFC, VMPFC, and DLPFC respectively. We specifically analyzed layer width, cell size, and cell density in order to determine differences of both total neuronal and interneuronal populations between areas. We also wanted to answer whether or not interhemispheric differences existed. Lastly, based on the assumption that the highly complex cognitive processing attributed to the DLPFC relies on functional and structural features of the layer II of this region, the discussion of our findings focused mainly on understanding Area 46 features. 

## Materials and Methods 

In the present study we used human postmortem tissue from five subjects, all males between the ages of 26 to 53 year-old. These subjects did not present evidence of SNC lesion according with the forensic evaluation. This material was obtained via the Instituto de Medicina Legal y ciencias forenses (Cali, Colombia). All procedures were approved by the Ethical Commites of the Universidad del Valle Health Faculty and Instituto de Medicina Legal y Ciencias Forenses de Cali, according with the Helsinki protocol. 

Vibratome Coronal sections of 50 µm were obtained from each cortical area (BA 46, 10, 24). Sections were immersed in 1.5% normal horse serum (Vectastain Ellite ABC, Vector Laboratories ®) in PBS for 40 min. Sections were incubated for 18 h with primary antibodies against the CBP, Parvalbumin, Calbindin, and Calretinin (PV: 1:500, CB: 1:5,000, CR: 1:2,500) to identify interneuronal populations. We also used an antibody against NeuN (1:250) to display the whole neuronal population. After incubation, sections were washed in PBS three times for 5 min each. Then sections were incubated in avidin-biotin HRP complex (Vectastain Elite ABC ®), with the respective secondary diluted at 1:200 for 40 min. The immunolabeling was developed in a substrate solution of 4% diaminobencidine, 2% hydrogen peroxide and 2% niquel diluted in PBS for 10 min. Finally, sections were washed in PBS and mounted in cromalumin slides for observation under the light microscope.

### Photographic register

Microphotographs were shot using a Cannon Power Shot A-430 camera (4.0 mega pixels), adapted to an Olympus CH-2 Optic light microscope (CHS model) at 10x. Photographic microns areas were selected. Coronal consecutive photographs (570X430), three from each subject, were taken from molecular layer to white matter. Images were fused using the Cannon Photo Stich version 3.1 2000 ® program in order to represent the whole cortical column. Measures and counting procedures were made using Sigma Scan Pro 5.0 program. Significant differences between the three analyzed areas of each hemisphere were determined applying ANOVA for repetitive measures. Then, differences were confirmed between two areas by using Student's t test. Interhemispheric differences between homologues areas were determined using also Student's t test. Statistical significance was set at *p* <0.05.

## Results

### Layer II Width

Areas 10, 24, and 46 showed layer II thickness differences when seen in each hemisphere. These differences were significant when using Anova as well as when pair comparisons were made using Student's t test. At statistical level, these differences accounted for the 50% of the total variance (*p *<0.001). None interhemispheric differences were found (*p >*0.05). 

### Neuronal density

**Figure 1.**
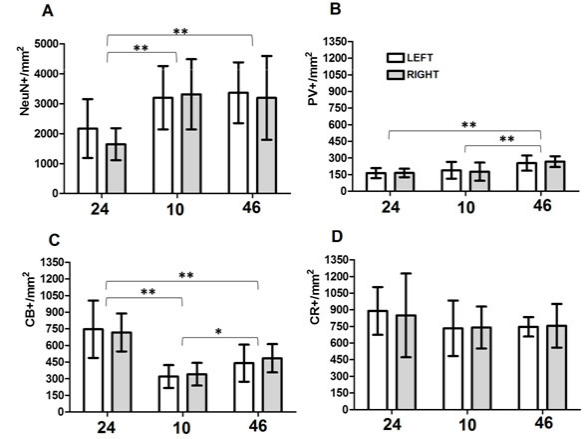
Layer II neuronal subpopulations density in human prefrontal areas 24, 10 and 46 correspond to cortical areas analyzed. Left: Left hemisphere. Right: Right hemisphere. NeuN+/mm^2^: density of neurons in layer II. PV+/mm^2^: density of PV-positive neurons in layer II. CB+/mm^2^: density of CB-positive neurons in layer II. CR+/mm^2^: density of CR-positive neurons in layer II. The mean values and the respective standard deviations are shown in the bar graph. *: statistically significant differences at *p *<0.05; **: statistically significant differences at *p *<0.01.

The three analyzed areas displayed differences in the total neuronal density when NeuN-IR cells were counted. These differences were significant as probed by ANOVA (*p *<0.001). These differences may explain the 35% of the total variance we found. However pair comparisons demonstrated that only differences between areas 24 and the other two ones were significant. Interhemispheric differences were not determined (Fig. 1). 

The three populations of interneurons (PV, CB and CR) showed significant differences in the following way: values for PV population (*p *<0.001) showed that differences between areas represent the 50% of the total variance. Pair comparison displayed that the origin of the variation corresponds to the differences in area 46 against the other two areas (Fig. 1).

Values for CB (*p *<0.001) showed that differences between areas explained almost 64% of the total variance. Pair comparisons displayed that all differences between areas are statistically significant (Fig. 1). 

Values for CR (*p >*0.05) showed that this analysis might not lead identify significant differences between areas, which could explain almost 8% of the total variance (Fig. 1).

### Cell soma area

**Figure 2.**
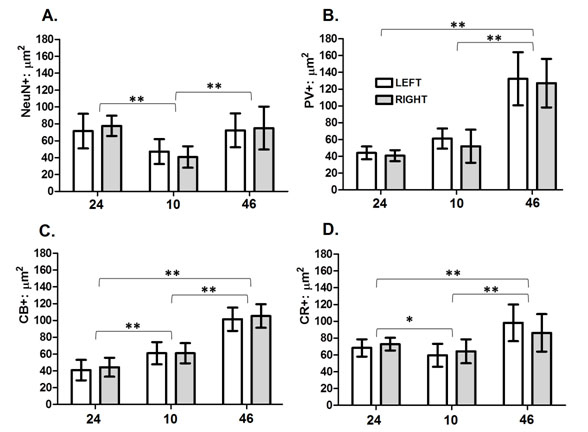
Layer II neuronal subpopulations soma size in human prefrontal areas 24, 10 and 46 correspond to cortical areas analyzed. Left: Left hemisphere. Right: Right hemisphere. NeuN+: µm^2^: soma size of NeuN-positive neurons in micrometers^2^. PV+: µm^2^: soma size of PV-positive neurons in micrometers^2^. CB+: µm^2^: soma size of CB-positive neurons in micrometers^2^. CR+: µm^2^: soma size of CR-positive neurons in micrometers^2^. The mean values and the respective standard deviations are shown in the bar graph. *: statistically significant differences at *p *<0.05; **: statistically significant differences at *p *<0.01.

The three analyzed areas showed significant differences in the soma area when NeuN-IR was evaluated and by using ANOVA. Nonetheless, these differences were significant as probed by ANOVA (*p *<0.001). These differences may explain the 35% of the total variance we found. However pair comparisons demonstrated that only differences between areas 24 and the other two ones were significant. Interhemispheric differences were not determined. This parameter analyzed according with the NeuN-IR does not showed significant differences between hemispheres in areas 24 and 46, meanwhile area 10 cell somas showed a significant higher size in the left hemisphere. Student t test did not allow identifies significant differences between hemispheres. However, the area value for the whole cell population (NeuN) showed significant differences in all three areas (*p *<0.001); which may explain almost the 41% of the total variance we found. Pair comparisons showed that the origin of the variance was due to the smaller cell bodies in area 10 when compared with the other two areas (Fig. 2). 

**Figure 3.**
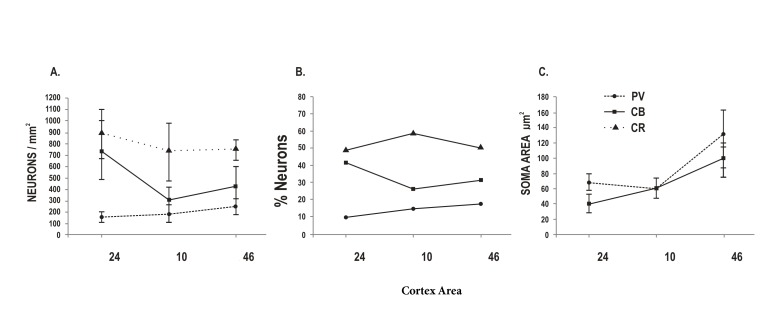
Comparison of neuronal density, proportion and soma size in layer II interneuron subpopulations of three human prefrontal areas 24, 10 and 46 correspond to cortical areas analyzed. PV: PV-positive neurons. CB: CB-positive neurons. CR: CR-positive neurons. Neurons/mm^2^: neuronal density. % neurons: percentage of neurons of a subpopulation of the total number of interneurons. Soma area µm^2^: soma size in micrometers^2^. The mean values and the respective standard deviations are shown in the graph. The filled circles, squares and triangles represent discrete values, and continuous and dotted lines show trends of change between cortical areas analyzed.

Additionally the ANOVA test showed significant differences between areas for each cell population analyzed as follow: PV values (*p *<0.001) showed significant differences, although it only accounts for the 22% of the total variance. However pair comparisons identified that the origin of the variation is due to the differences among area 46 with the other two areas (Fig. 3).

Calbindin values (*p *<0.001) showed that differences between areas account for the 81% of the total variance. Pair comparisons displayed that all areas were statistically different between them (Fig. 3).

Calretinin values (*p *<0.001) showed that differences between areas might explain almost 40% of the total variance. Pair comparisons displayed significant differences among all three areas (Fig. 3).

## Discussion

A progressive increase in cell density and "strengthening" of interneurons (particularly the PV-IR subpopulation) from Area 24, followed by Area 10 and ending up Area 46, represented the main results of the present study. First, we believe this pattern corresponds to the *archicortical trend* proposed by Yeterian *et al*.^1^, in which the current analyzed areas are found. 

On the other hand, our results also fit in previous models of the evolution of cerebral cortex derived from macaque and proposed by Dombroski *et al* 19. These authors stated that newest cortical areas tend to show an increase in layer width and total neuronal density. In addition, specifically talking about interneurons, newest cortical areas display increase in both the number of PV-IR neurons and soma size as well as decrease in the number of CB-IR cells. Subsequently Dombrosky *et al*. 19, categorized the area 24, which is located in the medial surface of the hemispheres, as an intermediate type between *"agranular"* and *"dysgranular"* cortices. Area 24 then represents the lowest level of the phylogenetic scale of primate PFC, indeed this area is found mainly processing limbic information. 

Moreover, Area 10, located at the pole of the frontal lobes, was classified as an intermediate type between *"dysgranular" *and *"eulamminated type I"* cortices. Thus Area 10 represents an intermediate level in the phylogenetic scale of PFC and functionally is found both dealing with both limbic and cognitive information as well as intrafrontal processing. Lastly, Area 46, located in the lateral surface of the hemispheres, (rostral portion of the middle frontal gyrus) was characterized by these authors as an "eulaminated type II" cortex, the highest phylogenetic level. In fact, area 46 is well known to be associated with information processing of high complexity in cognition such as abstract representations, planning, and spatial/temporal organization of behavior 19, 20.

In the present results, layer II of area 24 exhibited the smallest width when compared with the others analyzed areas. This width represented around 7% of the total cortical thickness. Layer II of this area also showed the lowest cell density, nearly 40% less, compared to areas 10 and 46. Furthermore, in regards of CBP-subpopulations, we observed that the highest density of interneurons corresponded to CB-IR cells (Fig. 3); nonetheless the cell area of these cells was significantly smaller than CB-IR neurons found in the layer II of areas 10 and 46. These dense but small CB-IR neurons might correlate well with the smallest width we found in the layer II of Area 24.

 Calbindin immunoreactive neurons, mainly located in supragranular layers, constitute 25% of the total interneuronal population in the primate PFC 21. Vertically oriented CB-IR neurons, better known as "double bouquet" cells (DBC), are thought to be the most abundant interneuron phenotype in the primate cortex 22. This type of neurons are found periodically distributed in the cortex and characteristically display a descendent axon with a dense ramification. This particular axonal system, also called *horse tail,* crosses the cortex from layer II to VI to produce vertical inhibition of pyramidal neurons placed in the same cortical column. Therefore, DBC are not only proper of primate cerebral cortex but also seem involved in maintaining cortical columns integrity 23, 24. On the other hand, the high density and small soma of CB-IR cells we found in layer II of area 24 is consistent to trans-laminar and vertical modular regulation on the whole width of the cortex. In contrast, modules restricted to layers II/III have been described for DLPC. 

Further, layer II of area 10 showed the highest laminar width, around 9, 2%. This result is consistent with the well-known highest width of the whole cerebral cortex in Area 10 when compared to others in the PFC. Moreover, CR-IR neurons in the layer II of Area 10 showed the lowest density and cell soma area between CBP subpopulations in each hemisphere. Nevertheless, these differences were not significant.CR-IR cells represent approximately 50% of all interneurons in the primate PFC. These cells with small somas appear mainly located in supragranular layers in close relationship with the pia surface 21.

 Morphologically, two types of CB-IR neurons are distinguished, *"bipolar"* and *"multipolar"* cells 25. Functionally, CB-IR neurons are found forming symmetric (inhibitory) synapsis in 93% of cases in the primate PFC. Since the axons of these cells are placed on the dendritic arbor of other interneurons, it is believed that CR-IR neurons are ultimately responsible of disinhibition of pyramidal cells located in supragranular layers. In contrast, synapses mediated by CR-IR neurons in the infragranular layers produce both inhibitory and excitatory effects 23, 26. 

Layer II of area 46 showed the highest cell density, 42% higher than area 24 and 1 % higher than area 10, though these differences were not significant. Regarding CBP, layer II of Area 46 showed the highest PV-IR cell density and the biggest soma size. We also observed that the size of the CBP subpopulations, particularly PV-IR ones, progressively increase as more rostral and lateral a specific analyzed area was. This pattern is also consistent with the evolution trends proposed by Dombrowsky *et al*. 19, as discussed previously. In consequence, layer II of Area 46 may be characterized by the presence of a strong interneuron system as compared to other areas, which in turn are mainly constituted by PV-IR cells. Further, taking into account that is known a wide and profuse distribution of processes of these interneurons; layer II of area 46 inhibitory activity, mediated by PV-IR cells, should expected to be wide and profuse as well. 

 parvalbumin immunoreactive neurons of the primate cortex constitute 25% of the whole population of interneurons. *"Basket"* and *"chandelier cells"* are the recognized morphological types which contact and affect the firing of pyramidal neurons. *Basket* cells are additionally classified in three subgroups depending on the size and the extension of the cell processes. *Small basket cells* (SBC) represent at least half of interneurons in supragranular layers and account for a high number of synapsis onto somas and proximal dendritic processes. The presence of electrical synapsis in this cell population has been also described. The inhibition associated to SBC exerts an important effect on properties such as synchrony and rhythm in the firing of pyramidal neurons which in the DLPFC may result in SF 23, 27.

Chandelier cells (CC) are mainly located in layers II/III. These interneurons are vertically oriented and exhibit multiple synaptic contacts especially onto the axon initial segment of pyramidal neurons. It is estimated that 35-50% of the pyramidal population is contacted in the range of the distribution of the CC axon. Indeed, a synaptic ratio of 1 pyramidal to 4 CC is proposed. These characteristics lead to postulate that CC synapsis may be the most powerful inhibitory system of the cerebral cortex 23, 28. Moreover, in addition to the inhibitory effect, CC have seen inducing depolarization on pyramidal neurons and thus generating an excitatory response. The excitatory or inhibitory responses of CC may depend on which firing state the specifically influenced neuronal network is 29.

Furthermore, we did not determine differences between hemispheres when homologues areas were contrasted. Nevertheless, functional interhemispheric differences (FID) have been extensively described. First, it is possible that different types of information is segregated and processed in a hemisphere-specific way without relying on structural differences. Further, FID may be placed at the level of networks and circuits, which might be recruited at task convenience; structural differences may also not be requested. Lastly, FID may rely on molecular changes that are not under the scope of this work.

Ultimately, the diversity of patterns of interneurons as well as the modular organization of supragranular layers (especially I, II and IIIa) in the layer II of Area 46 may constitute the basis for SF. In summary, layer II of area 46 as compared to other prefrontal areas is characterized by a powerful system of inhibitory interneurons that are also thought to function via self-regulation activity. Furthermore, this system appears organized as a modular architecture in supragranular layers. From a physiological point of view, SF, as a result of the performing of these modules, is considered the basic mechanism of the functional phenomenon of resistance to interference, which is characteristic of the DLPC 30, 31.

In this context, the specific cortical modules of layer II of area 46 are expected to raise and sustain an online representation of information of high complexity. This function might be consequence of the interaction of small pyramidal neurons and interneurons (particularly chandelier cells). Excitatory afferents to these modules are expected mainly to come from other prefrontal areas (9/46,45 and 8), which in turn are already receiving information from sensory association cortices located in the post-rolandic cortex 32- 34. This phenomenon could be the basic mechanisms of complex cognitive processes such as working memory, cognitive control, selective attention and decision taking 14, 35- 38.

## Conclusion

Layer II of area 46 possess a powerful inhibitory system as compared to other prefrontal areas. This inhibitory system will regulates the prefrontal sustained activity during working memory. The interneuron subpopulations activity defined the modular architecture of supragranular layers.
